# Continuity or Change: Occupational Health Policies of Key Organizations in Great Britain After the Major COVID Waves 2020-2022

**DOI:** 10.1177/10482911251391853

**Published:** 2025-11-10

**Authors:** Andrew Watterson, Matthias Beck

**Affiliations:** 1 Faculty of Health Sciences and Sport, 7622 Stirling University, Stirling, Scotland; 2 Department of Management & Marketing, 8795 University College Cork, National University of Ireland, Cork, Ireland

**Keywords:** Covid-19, occupational health and safety regulation, enforcement, Great Britain

## Abstract

The neglect of occupational health and safety within Great Britain spans many decades. There has been limited or no recognition of many occupational diseases partly compounded by a chronically under-funded and under-staffed central and local government inspectorate. Workplace inspections have declined. Little enforcement, prosecution and fines of those breaching laws occur. With its departure from the European Union, Great Britain has further neglected hazards at work and has become even more committed to soft regulation and de facto deregulation under the guise of “better regulation” to support economic growth. The Covid pandemic highlighted many workplace health and safety shortcomings. With few exceptions - notably non-governmental organizations, trade unions and some professional bodies - relatively little appears to have been learnt by government and regulators in terms of policies and practices that will improve worker health and safety post-Covid and avoid similar disasters in future pandemics.

## Introduction

Prior to the COVID-19 (COVID hereafter) pandemic, occupational health and safety across the three nations in Great Britain (GB)—England, Wales, and Scotland—was neglected by governments over several decades: occupational health perhaps more so than occupational safety.^1–8^ The COVID pandemic has highlighted the importance of occupational health and safety on numerous levels making it worthwhile to map the post-2022 GB occupational health policy terrain with a view toward identifying potential impacts of the pandemic on relevant policies of successive GB governments.

The United Kingdom (UK) refers to England, Scotland, Wales, and Northern Ireland. GB refers only to England, Scotland, and Wales. The worker health and safety regulatory body, the Health and Safety Executive (HSE), covers England, Scotland and Wales: a separate body covers Northern Ireland. The article does not address occupational health and safety in Northern Ireland.

However, within GB, local authorities and not the HSE have regulatory, inspection, and enforcement roles for retail, wholesale distribution, warehousing, hotel and catering premises, offices, and consumer and leisure industries. These workplaces were all critical during the various COVID waves in regulatory efforts to stop the spread of the disease.

The position becomes more complex when examining the laws, policies, and regulations related to health and COVID controls in workplaces and their impacts during the pandemic. The UK Department of Health and Social Security, despite its title, covered health and social care only in England including COVID vaccination policies in English workplaces. Within GB, separate health departments covered health and vaccination policies in Scotland and Wales. The UK Department of Work and Pensions similarly covered only England with regard to various financial support and furlough schemes for those laid off due to COVID cases, but it did cover all GB regarding recognition or nonrecognition of COVID as a prescribed occupational disease and state payments for those with recognized occupational diseases.

GB occupational health policies prepandemic were meant to involve input from government, employers and employees. The complexity of interactions among these three interest groups was reduced by the fact that GB Governments largely tended to ignore workers and trade unions when they supported stronger regulation. Nonetheless, occupational health policies and practices varied among employment sectors, regulators, and workplaces. The COVID pandemic involved additional bodies from public health in workplace health and safety. The UK's 2020 Coronavirus Act, which enhanced governmental powers to slow the spread of the virus,^9^ provided powers to the Scottish government and Northern Ireland Executive to pass “lockdown” regulations, and to consult with trade unions on guidance to control COVID in the workplace.

The major occupational health policy problems that emerged during the pandemic relate to interpretation and enforcement of laws. The fitness for purpose of UK legislation on occupational health and safety in general has been disputed.^10–12^ There is, however, some consensus about past failures by Governments and the HSE, with respect to the implementation of regulations.^13–15^ In 2015, some GB occupational physicians criticized the HSE for failing to fulfill its statutory duties to protect workers from occupational diseases. They noted that “the ethos of the 1802 Factory Act—that the state has a duty to protect workers … —has been reversed. Doctors working in occupational medicine are beholden to employers, who have little incentive to find or prevent occupational diseases.”^7^

Since the 1980s, UK Governments adopted increasingly neoliberal approaches to occupational health and safety, viewing it as a source of unnecessary “red tape” constraining economic growth and supported by European Union bureaucracy. The ramifications of this approach have been explored by occupational physicians^7^ and in analyses of UK corporate crime.^16^ Brexit worsened this situation as the UK Government pushed its deregulatory agenda further, while the main GB regulator, the HSE, remained understaffed and underfunded, and its leadership felt obliged to reflect ideological positions of the Post-Brexit UK Governments.

According to HSE official figures (that significantly underreport occupational ill-health), in 2021/2022 there were 1.8 million working people suffering from work-related illnesses including 722,000 new cases of work-related ill-health each year. A total of 13,000 cases of occupational disease were estimated to occur each year primarily due to chemical and dust exposures.^17^ UK COVID mortality figures, based on Johns Hopkins data of December 2022, totaled 118,382 deaths (312.70 per 100 000) which exceeded corresponding figures for France, Spain, Sweden, and Germany.^18^ Workplaces played a significant part in virus transmission. Within GB, Scottish COVID mortality figures were particularly poor for reasons that are not yet fully clear.

Three factors contributed to the United Kingdom's high COVID mortality figures:

1. Control measures built into government and governmental “COVID-secure” guidance (covering planning, social distancing, masks, ventilation, cleaning, testing, and contact tracing) were often inadequate and sometimes wrong.
2. Employer working practices, planning, and risk assessments were often poor.
3. Monitoring, inspection, and enforcement by the health and safety regulators—the HSE and local authority health and safety inspectors—were sometimes deficient. Relatively few data were publicly available during the early stages of the pandemic.^19–23^ However, there are indications that occupational health risks faced by GB workers both from the pandemic and preexisting workplace hazards were considerable and unequally distributed across the population; precarious workers, migrant workers, essential health, social care and public sector workers, key workers in transport, and food production and supply, were particularly exposed.

Lasting up to 2022, the COVID pandemic had multiple waves over three years. Addressing occupational health challenges successfully would have required the ongoing application of rigorous approaches that were known to work globally. Such approaches had long been advocated by bodies like the ILO and European regulatory agencies who typically adopted a “hierarchy of risks” approach. This involved an emphasis on nonpharmaceutical interventions (NPIs) throughout the pandemic when vaccines did not exist, and later, when vaccines did emerge, using NPIs to maintain successful preventive measures. This was in line with the precautionary principle, prevention priorities, and the recognition of COVID as an occupational disease. It included a focus on ventilation in the workplaces and the full use of the best available personal protective equipment.^24^ In addition to major steps toward effective vaccine development to combat variants and measures relating to testing and tracing, most of these pandemic measures emphasized lockdowns, isolation, restricted movement of workers, economic support for employers, and economic support for workers losing income or suffering from COVID and long COVID. These measures could all have been viewed as integral parts of good and established occupational health and related policies and practice.

Official government and regulator assessments of their work around COVID contrasted sharply with some of the findings of several parliamentary and departmental committee reviews tasked with oversight and scrutiny of both governments and regulators. This has become particularly apparent following the UK COVID Inquiry, which began holding public hearings in June 2023. Meanwhile, responses and policies of various GB departments and bodies indicate that there were high levels of heterogenicity of challenges and practices. Preliminary evidence indicates that the occupational health challenges employers and employees faced during the pandemic in the health service, care sector, transport, construction, meat plants and call centers were among the greatest.

Notwithstanding flawed government policy, UK nongovernmental organizations (NGOs), consistent with their advocacy and campaigning roles on health and safety, have provided detailed analyses as well as extensive proposals to take occupational health forward during and after the pandemic. This has included guidance and analyses suggesting changes needed to address COVID threats during the various waves. Several organizations have suggested improvements to occupational health services in the future and recommended engaging more directly with trade union safety representatives in particular workplaces. International bodies like the ILO and the WHO initially had limited GB impacts. This was so even though they had identified some of the occupational health threats prior to the pandemic and had highlighted the need to plan for such occurrences. In most respects, successive GB governments had failed to address such planning issues. The WHO's own lack of attention to certain risks arising from poor PPE, inadequate ventilation and lack of physical distancing was, meanwhile, often mirrored by actions of GB bodies.

## Government(s) and Parliament

The UK Parliament debates legislation and policy, and records witness statements for reports appearing in the public domain. It does, however, have relatively little impact on changing policies and practices on occupational health. The Welsh and Scottish Parliaments develop most of their own public health policies within UK Government financial constraints.

Recent UK Government policy has embraced neoliberal thinking with a focus on soft regulation, better regulation and deregulation agendas to aid industry and other employers: the so-called attack on “red tape.” These hostile attitudes were applied repeatedly to occupational health and safety policies and regulations and proved characteristic of many of GB governments’ responses to the pandemic waves. Data collected during the later waves of the pandemic, indicate that these attitudes have continued to shape UK planning on occupational health.^25^ Even in areas where specific policies were found to work, the UK Government still pushed deregulatory campaigns.^6^ In 2018, an in-house review by the Department of Work and Pensions^26^ of the HSE, nonetheless, “called on the safety regulator to up its game, echoing concerns raised by the GB Trades Union Congress, individual trades unions and safety campaigners.”^27^

The establishment of the NHS in the 1940s never created a national occupational health service in the United Kingdom. Subsequent legislation, including the 1974 Health and Safety at Work, etc, Act, which established the Employment Medical Advisory Service, did not fully rectify this. The United Kingdom's neoliberal policy trajectory eventually culminated in the referendum on the withdrawal from the EU. With Brexit, new measures to reduce “red tape” have been introduced which included a 2022 Government proposal to drop a raft of EU-based regulations that on paper protect the occupational health of workers. This included proposals to exempt more than 40,000 businesses from reporting requirements and regulations on health and safety.^13^

According to official figures, the impact of COVID on GB occupational ill health was very dramatic. During 2021/2022, an estimated total of 123,000 workers suffered from COVID which they believe may have been due to coronavirus exposure at work. Excluding these, 123 000 COVID cases, in the same year, 585 000 workers recorded having had work-related illnesses caused or worsened by COVID (new or long-standing).

GB Government departments, largely failed to exploit the potential of existing law for protecting occupational health effectively during the pandemic. This was recognized by occupational health professionals, researchers, trade unions and NGOs as early as 2020.^10,19,20,28^ There were “notable failures” in health and social care and in education, linked to ignoring the precautionary principle, conducting faulty risk assessments, inadequate enforcement and/or insufficient provision of PPE.^10^ Similar regulatory failures existed prior to the pandemic.^5,11,15,20–23^

Research conducted in June 2021, indicated that mask upgrades would have cut infections. *Hazards* and the British Occupational Hygiene Society (BOHS), the Society of Occupational Medicine and trade unions all called for PPE standards to be raised from about 2020 onwards. Yet, appeals to the Scottish Government to do so were consistently ignored.^29^

## Regulators

The HSE is an executive nondepartmental GB regulatory public body responsible for inspecting and enforcing health and safety laws in most but not all workplaces. During the pandemic, there were no major new laws for the HSE to enforce but there were some important shifts in the direction of the organization driven by its leaders.^30,31^ These encompassed the further softening of the inspection and enforcement messages to employers and employees alongside a movement away from tripartism (with reduced involvement of worker organizations at board level).

Officially, the HSE accepts there is a challenge addressing occupational ill-health and wishes to reduce worker ill-health. Yet, its business plan for 2022/2023 contained no practical details on how to achieve this. HSE leaders highlight its role as “a proportionate and enabling regulator” and state they want to increase and maintain trust in what it does.^31^ However, the House of Commons and NGOs found that the HSE as regulator went missing during the Pandemic, a time in which it inspected only 1 in 218 workplaces. It lost trust due to its failure to enforce.^28,32,33^ The HSE's actions on occupational health threats did not look preventative but the HSE 10-Year strategy up to 2032 again refers to a refreshed set of priorities, including mental health and stress.^34^

HSE's leadership was criticized in parliamentary inquiries and by NGOs for using poorly trained private contractors to conduct COVID workplace risk assessments. An all-party House of Commons Committee noted in June 2020 that during the pandemic to date the HSE had: “required just one business to close. It did not inspect a single care home since 10 March 2020. However, without records of the number of businesses that have closed voluntarily after an intervention by HSE, it is impossible to get a clear picture of the impact its work has had.”^35^ The all-party House of Commons committee further noted that the HSE had conceded that

the number of occupational deaths it has recorded through RIDDOR reporting (Reporting of Injuries, Diseases and Dangerous Occurrences Regulations 2013) is likely to be significantly lower than the reality, particularly in NHS settings. We are not persuaded that its efforts to tackle under-reporting have gone far enough or fast enough. In early June, it was still working on a new guidance.^35^

Local authorities in England, Wales, and Scotland are large employers of inspectors responsible for enforcing workplace health and safety legislation in certain workplaces. They cover retail, wholesale distribution and warehousing, hotel and catering premises, offices, and the consumer/leisure industries. They do not have the same wider functions or capacity as the HSE in terms of research and publication on occupational health issues, although they produce codes and circulars to inform the employers they inspect. Local authorities often liaise closely with HSE.^36^ Together they are meant to manage the health and safety of their workforce and those affected by their work. Local authority inspectors were possibly more active than the HSE during the early stages of the pandemic in enforcement actions.^28,37,38^

## Employers

GB employers are heterogeneous and include multinationals as well as small contractors and self-employed subcontractors. During the 1990s, several larger umbrella bodies accepted the HSE's evidence from the 1990s on the economic burden of poor health and safety for employers. From 2010 onwards, however, they actively pressed the HSE to improve GB occupational health. The Confederation of British Industry (CBI) recently called for modernization of GB's Occupational Health services to reduce workforce disease burden, partly in response to the impact COVID had on the occupational health of the workforce. In the CBI's own words, they were campaigning to improve health standards for the working population^39^:

Health is a key enabler of social and economic progress. Businesses are recognising that early and sustained support through workplace health interventions are an effective way to minimise the risk of job losses related to ill-health. But support doesn’t just benefit business. Improving workforce health impacts both NHS costs through the disease burden, as well as economic output from an increasingly productive workforce with reduced sickness absences.^39^

How this will translate into action among their members is less clear.

Specific industry interest groups like MAKE, formerly known as the Engineering Employer Federation (EEF), have a long record of surveys on worker health and safety in their sector and have supported occupational health. They have published policies^40,41^ with a focus on training and guidance. In 2017, the UK Government produced an industrial strategy that omitted any reference to the need to have a fit, healthy, and productive workforce. For MAKE, this was a missed opportunity.^40^ In 2018 the EEF joined the call from the Society of Occupational Medicine for universal access to occupational health.^40^ The Federation of Small Businesses also provided resources for its members on general health and safety policies, but nothing specific on occupational health.^42^ It did, however, provide advice on COVID workplace measures.

## Professional Bodies

Bodies representing occupational health and safety professionals of course focus on occupational health and its development. Most offer training courses and publish journals. The Society of Occupational Medicine (SOM) and the BOHS were assessing the state of occupational health and offered recommendations for future improvements before and after the pandemic. The SOM has produced substantial analyses and reviews on the importance of occupational health^43^ and highlighted the possible links among health, work climate, and sustainability.^44^ Nicholson synthesized past evidence for the business case for investment in occupational health in terms of legal, moral and financial drivers that influence employers.^43^ SOM, in a Royal College of Physicians (RCP) publication, on COVID highlighted the need for occupational health services to act as a bridge between health and workplaces^44,45^ has identified the need for future measures to protect worker health that relate to designing safe and sustainable work environments with cleaner air.

The Faculty of Occupational Medicine (FOM), within the RCP, is one the UK's main professional bodies for occupational medicine with 1800 members. Some of these members work in the NHS, are researchers and educators, or offer consultancy services to businesses. The Faculty produces publications and guidance.^46^ FOM has a “Safe Effective Quality Occupational Health Service” scheme, which includes an accreditation system for health services. Its journal, Occupational and Environmental Medicine, contains occupational health policy papers which do not necessarily come from FOM or relate to GB.

In 2020, two FOM Scotland occupational physicians issued a manifesto for Occupational Health.^47^ This related to improving access to occupational health services across Scotland. The manifesto also demanded more investment in NHS services as well integration between occupational health and public health and the development of a dedicated Scottish Center for Occupational Health.

A BOHS strategy emerged in 2021 during the pandemic, and the organization plans to develop this up to 2025. It aims to eliminate harmful exposures and design out threats to human health.^48^ During the pandemic, BOHS supported interventions on raising PPE standards for exposed workers.^29^

A professional body representing local authority inspectors, the Chartered Institute of Environmental Health, provides information on specific occupational health issues affecting its inspectors. It also provides general information about occupational health, runs courses and offers advice to employers on preventing occupational ill-health. However, it appears to lack resources to review policies.^49,50^

The Institution of Occupational Safety and Health is the largest professional occupational health and safety body in the UK with around 48 000 members. Many are workplace health and safety advisors employed by businesses. The organization offers advice on occupational health subjects and funds research as well as input in UK Government consultations. It also hosts an extensive training program including webinars on COVID and relevant tools such as risk assessment.^51^

## Trade Unions

Trade unions have long engaged in campaigning activities in occupational health and safety. The TUC is the umbrella body for most trade unions in GB. It has produced a range of policy, documents and education programs that include occupational health. These are complemented by the Risks newsletter which provides up to date information about occupational health issues from a UK and international perspective.

During the pandemic, the TUC's focus was on supporting safety representatives, preventive measures, and the recognition of COVID and Long COVID as an occupational disease. The information provided to its reps appears to seek to take occupational health forward.^33,52^ The TUC has produced guides and reports on such topics as risk assessments, safety inspections, occupational cancer, occupational hygiene, protecting health and safety after Brexit, and Registration, Evaluation, Authorisation and Restriction of Chemicals.^53^

The pandemic may have increased the intensity of trade union occupational health activity, but not necessarily its scope. Key policies already existed that these unions viewed as necessary for dealing with any biological hazards including COVID and bird flu. Two unions illustrate this:

GB's second largest union, Unite (which represents workers across construction, manufacturing, transport, logistics, and other sectors), produced generic guides on occupational health and safety with some material specifically relating to COVID. In addition, they provided educational courses on occupational health and published specific guides on occupational health issues such as dust, asbestos, diesel fumes, plastics and composites local exhaust ventilation, foundry chemicals and 3-D printing.^54^ Moreover, the union addressed specific problems created by the pandemic and pressed for greater action and better risk assessments on ventilation, PPE, and HSE inspections and enforcement.

Unison is GB's largest union with almost 1.4 million members (mainly from the public sector). It has a dedicated national web page on occupational health.^55^ The union supports its branches on occupational health issues and provides checklists relating to the occupational health services needed, occupational health unit programs and targets together with instructions on type and content of health assessments. Some unions like the Royal College of Nurses (RCN) and British Medical Association (BMA) play a professional role in occupational health within the NHS, and employers working within commercial occupational health companies.

The British Medical Association (BMA)^56^ represents all GB medical practitioners and has a well-established Occupational Medicine Committee that has been very active during the pandemic, providing information and challenging GB Governments and NHS occupational health policies. The BMA proposed improved policies on testing, risk assessments, PPE and infection control both for health workers and patients.^57^

The RCN has its own health and safety advisors and represents the professional interests of occupational health nurses. It also provides occupational health advice including information about COVID to its members as well as participating in various committees, consultation exercises and campaigns.^58^ It draws on information from HSE and bodies such as Public Health England.

Most UK unions postpandemic have not produced new or amended occupational health policies that reflect or address many of the problems that emerged during the pandemic. Their major channel for shaping future occupational health policies beyond their education programs and publications involves bargaining in workplaces and consultations with government organizations. Evidence suggests that these consultation exercises have little impact on GB cabinet policies.

## NGOs and Health and Safety Campaign Groups

NGOs, like employer and trade union bodies, cover a wide range of interests and are very diverse in terms of policy objectives. In the UK, the established Hazards Campaign and the linked Hazards Magazine focused on worker and community groups and discussed possible future developments in occupational health. They are both campaigning bodies and sources of information, substantial analysis and manifestos.

The Hazards Campaign, with its sister organization Scottish Hazards, has a long history of policy advocacy on workplace occupational health. During the pandemic, the Campaign raised a wide range of issues with the HSE about regulation based both on general 2019 policy objectives and particularly about how existing health and law was being interpreted by HSE. The Campaign was especially worried about the fact that HSE's designation of COVID was downgraded to merely a “significant” workplace risk, rather than a “serious” workplace risk—a classification which made enforcement and other regulatory actions weaker.

The 2019 Hazards campaign produced a manifesto for the general election with objectives they continued to advocate through 2022.^59^ The manifesto included a call to ensure a multidisciplinary, worker oriented, free NHS occupational health service available to all groups of workers, full and part-time, temporary and permanent, as soon as they started work in any sector. This was linked to a call for fair and just compensation for workers hurt or made ill by work would have involved changes in how the Industrial Injuries Advisory Council worked. The campaign additionally wanted to see a United States style whistleblower system on occupational safety through a hotline and protection unit within HSE.

The 2021 and 2022 Hazards Campaign priorities included support for implementing the ILO's fundamental human right to safe and healthy work in efforts to move to a society that prioritizes health, safety and dignity of its workers with protection from all public health risks. This covered biohazards, epidemics and pandemics. There were also calls for

continuous assessment and improvement of health, safety and employment legislation, to ensure that prevention of risks, is increased to the highest practicable standards.^59,60^

The Royal Society for the Prevention of Accidents (ROSPA) is a very different organization than the Hazards campaign. It does, however, have roles that include campaigning on a wide range of safety topics and on occasions addresses workplace health. A key ROSPA function relates to the provision of education courses. Its courses lead to qualifications for IOSH and NEBOSH certificates and include occupational health and risk assessment.

ROSPA, like the British Safety Council which also has a magazine that covers occupational health issues, does not appear to have a specific policy on occupational health However, in the 2010s, it recognized that “for too long occupational health has been the ‘poor cousin’ in the H&S relationship, even though work related occupational health damage is a far greater problem than occupational accidents.”^61^

The Institute of Employment Rights, a think tank for the trade union movement,^62^ has produced detailed analyses of occupational health and safety matters. These analyses contained proposals for addressing occupational health problems that emerged during and before the pandemic.^12^ In February 2021, IER's analysis of the HSE COVID during the Pandemic led it to conclude that there had been regulatory failure that required urgent actions.^25^ These actions were to include the holding of an independent public inquiry into the future of UK health and safety regulation, greater investment in HSE, a review of enforcement strategies, and stronger trade union rights. The latter was to entail enhanced rights to access workplaces, rights to undertake preventive work and issue improvement notices, greater information for and consultation with safety representatives. IER recognized the need to reform the laws to better protect workers in casualized employment including those in the gig economy of the future.

## Conclusions

As GB COVID cases and deaths began to climb back up again in 2023, it became clear that the earlier COVID pandemic waves had reflected preexisting and long-standing failings in GB occupational health policies that operated before 2020 and continue. These included multiple failures to obtain and use evidence, and utilize tools available to address occupational disease risks, as well as poor planning, poor risk assessments, lack of effective engagement with employees, low prioritization of prevention and precaution. In the COVID context, this manifested itself in the neglect of improved ventilation and effective PPE, poor communication and mixed messages. Some of these failures were compounded by political and regulatory anti red-tape rhetoric, and an absence of monitoring and enforcement by the HSE.

Unintended consequences of the pandemic, however, have emerged that may ultimately lead to changes and benefit occupational health in a “postpandemic world.”^63^ Firstly, there is now a wider recognition of the crucial role workplace health safety plays in protecting public health. This has stretched beyond the recognition of the critical role of healthcare workers who, if ill, cannot protect the population at large to, for example, bus and taxi drivers, shop workers, food processing workers, office workers, education staff, police and fire firefighters, and building and manufacturing workers. It also involved the recognition of the need to pay more attention to workplace risk assessments, to PPE, to working conditions, especially ventilation.

Today, there is greater understanding of the importance of WHO and ILO information and guidance on occupational health. The WHO's approach on prioritizing some groups of workers for vaccines and using principles of equity and reciprocity to guide decisions was well argued but neglected in GB.^22,23^ In addition, the ideas around the application of the precautionary principle and removing hazards at source in the hierarchy of risk controls have now become more prominent in public discourse. The pandemic has highlighted generic GB failures to address the need for official recognition of more occupationally caused and occupationally related diseases and to do so more quickly and compensate those affected adequately. The problems faced by those employed in healthcare and other workers suffering from long COVID have drawn attention to some of these issues.

Other consequences of the pandemic have emerged in the context of the governance of the GB nations: Scotland and Wales. Although occupational health and safety is a matter reserved to the UK parliament, the abrogation by HSE of the lead COVID role to public health agencies and indeed HSE's early absence from checks on COVID safety in workplaces meant that Scotland and Wales drew up their own workplace health and safety advice. Sectoral guidance was produced by public health bodies at times in conjunction with trade unions and independent sources. This fitted in with a “fair work” agenda that the devolved governments had already adopted prior to the pandemic, although too often little attention was paid to worker health, safety, and the wider environment in their reports.^20,21^ Zoonotic threats, sustainability, and climate change have now attracted more attention more attention in occupational health settings. These include efforts to create green jobs, application of toxic use reduction principles, and the use of trade union safety reps to support and advise workers in nonunionized workplaces on workplace occupational health issues.

Whether these positive initiatives to improve UK occupational health will survive any length of time is debatable. UK House of Commons committees investigated inspection and enforcement failings of the HSE—so critical to protecting occupational health—during the pandemic. Their lack of staff and deficient resourcing in dealing with COVID as an occupational disease stand out as key factors of wide-spread weakness.

While not directly addressing the role of the UK's weak and flawed occupational health and safety policies, the first part of the UK COVID Inquiry offers some telling observations about the state of public health:

The cost, in human and financial terms, of bringing COVID-19 under control has been immense. Government borrowing and the cost of procurement and of the various job retention, income, loan, sick pay and other support schemes have severely impacted public finances and the UK's financial health. The impact on the NHS, its operations, its waiting lists, and on elective care has been similarly immense. Millions of patients either did not seek or did not receive treatment and the backlog for treatment has reached historically high levels. Societal damage has been widespread, with existing inequalities exacerbated and access to opportunity significantly weakened.

Ultimately, the UK was spared worse by the individual efforts and dedication of health and social care workers and the civil and public servants who battled the pandemic; by the scientists, medics and commercial companies who researched valiantly to produce lifesaving treatments and ultimately vaccines; by the local authority workers and volunteers who looked after and delivered food and medicine to elderly and vulnerable people, and who vaccinated the population; and by the emergency services, transport workers, teachers, food and medicinal industry workers and other key workers who kept the country going.^64^

Devolved administrations have also established inquiries into COVID that should lead to better linked and coordinated policies on both occupational and public health with stronger interagency working between regulators like HSE, local authorities and environmental agencies on issues such as air pollution. Some have introduced bills that would improve disease recognition but still missed many opportunities to better protect workers at the time and plan for the future just as the WHO did.^65–67^ However, commitments in the past to occupational health have not overridden the neoliberal, deregulatory ethos and practice of UK Governments and their post-Brexit hostility to regulation.

The election of a new Government in 2024 was preceded by a Labour Party policy document on “making work pay.” This contained a small number of welcome references to occupational health and safety, but it did not provide a coherent and comprehensive pathway for improving occupational health across GB.^68^ There were promises to review health and safety guidance and regulation along with a mix of promises to address, for example, working temperatures, the plight of agency workers, and the need to promote well-being and mental health. Time will tell if effective actions follow from this government. Labour's landslide victory followed 14 years of Conservative rule. While Labour's overwhelming majority would, in theory, allow for a revival of occupational health and safety in Britain, the party's recent history indicates that progress in this area is not guaranteed.
